# Computational Basis for On-Demand Production of Diversified Therapeutic Phage Cocktails

**DOI:** 10.1128/mSystems.00659-20

**Published:** 2020-08-11

**Authors:** Catherine M. Mageeney, Anupama Sinha, Richard A. Mosesso, Douglas L. Medlin, Britney Y. Lau, Alecia B. Rokes, Todd W. Lane, Steven S. Branda, Kelly P. Williams

**Affiliations:** a Sandia National Laboratories, Livermore, California, USA; Dalhousie University

**Keywords:** *Pseudomonas aeruginosa*, bioinformatics, phage therapy

## Abstract

New therapies are necessary to combat increasingly antibiotic-resistant bacterial pathogens. We have developed a technology platform of computational, molecular biology, and microbiology tools which together enable on-demand production of phages that target virtually any given bacterial isolate. Two complementary computational tools that identify and precisely map prophages and other integrative genetic elements in bacterial genomes are used to identify prophage-laden bacteria that are close relatives of the target strain. Phage genomes are engineered to disable lysogeny, through use of long amplicon PCR and Gibson assembly. Finally, the engineered phage genomes are introduced into host bacteria for phage production. As an initial demonstration, we used this approach to produce a phage cocktail against the opportunistic pathogen Pseudomonas aeruginosa PAO1. Two prophage-laden P. aeruginosa strains closely related to PAO1 were identified, ATCC 39324 and ATCC 27853. Deep sequencing revealed that mitomycin C treatment of these strains induced seven phages that grow on P. aeruginosa PAO1. The most diverse five phages were engineered for nonlysogeny by deleting the integrase gene (*int*), which is readily identifiable and typically conveniently located at one end of the prophage. The Δ*int* phages, individually and in cocktails, killed P. aeruginosa PAO1 in liquid culture as well as in a waxworm (Galleria mellonella) model of infection.

**IMPORTANCE** The antibiotic resistance crisis has led to renewed interest in phage therapy as an alternative means of treating infection. However, conventional methods for isolating pathogen-specific phage are slow, labor-intensive, and frequently unsuccessful. We have demonstrated that computationally identified prophages carried by near-neighbor bacteria can serve as starting material for production of engineered phages that kill the target pathogen. Our approach and technology platform offer new opportunity for rapid development of phage therapies against most, if not all, bacterial pathogens, a foundational advance for use of phage in treating infectious disease.

## Abstract (precis)

The antibiotic resistance crisis has led to renewed interest in phage therapy as an alternative means of treating infection. However, conventional methods for isolating pathogen-specific phage are slow, labor-intensive, and frequently unsuccessful. We have demonstrated that computationally identified prophages carried by near-neighbor bacteria can serve as starting material for production of engineered phages that kill the target pathogen. Our approach and technology platform offer new opportunity for rapid development of phage therapies against most, if not all, bacterial pathogens, a foundational advance for use of phage in treating infectious disease.

## INTRODUCTION

The emergence of antibiotic-resistant bacteria has become increasingly problematic in recent years. A number of contributing factors have been identified, including overuse of antibiotics in medicine and agriculture, and reduced rate of discovery and production of new antibiotics (1). New treatments for bacterial infections, particularly those caused by antibiotic-resistant pathogens, are needed to combat the crisis. Phage therapy is an attractive alternative to antibiotics that is undergoing a revival (2–4) in the United States for this reason.

Bacteriophages are viruses that infect bacterial species, and they are regarded as the natural predators of bacteria (5). For use in treating bacterial infections, phages hold numerous advantages, including vast natural diversity, low cost, high specificity (such that microbiomes are left intact), and proliferation *in situ* (amplifying and sustaining therapeutic effects) (6). Therapeutic cocktails are generally composed of virulent phages (capable only of the lytic life cycle) isolated from environmental samples. Temperate phages (capable of both lytic and lysogenic life cycles) have typically been excluded because the resulting lysogenic target bacteria would survive to spread resistance to that phage; furthermore, temperate phages may carry cargo genes that promote antibiotic resistance and/or bacterial pathogenicity (7, 8). However, there are far more genome sequences available for temperate phages (in the form of prophages integrated within bacterial genome sequences) than for virulent phage genome sequences at NCBI (9), and temperate phages can be converted into nonlysogenic phages using modern genome engineering tools (e.g., by knocking out the integrase gene) (10).

**FIG 1:**
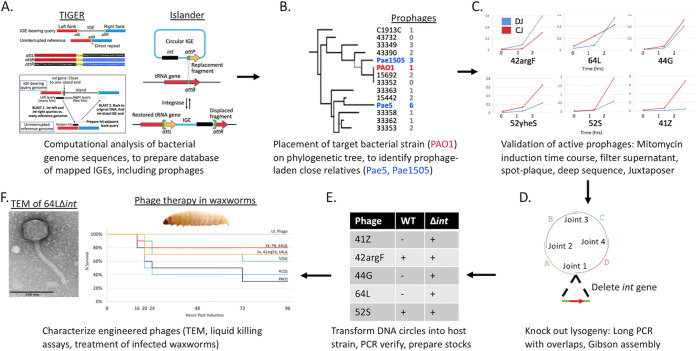
Pipeline for therapeutic phage cocktails. (A) TIGER and Islander algorithms are run on all sequenced bacterial genomes, yielding a database of IGEs, including prophages. (B) The target pathogen (PAO1) is placed on a phylogenetic tree in the database, such that close relatives bearing multiple prophages can be identified. (C) Prophage-laden strains are treated with mitomycin C, deep sequenced, and analyzed with Juxtaposer in order to identify prophages capable of mobilization. (D) PCR primers are designed to generate overlapping long PCR amplicons that rebuild the prophage genome without its integrase gene. The amplicons are joined using Gibson assembly, generating a circular Δ*int* phage genome. (E) The circular Δ*int* phage genome is transformed into a host strain (target pathogen or close relative), plaque purified, and verified by PCR. (F) WT and Δ*int* phages are characterized through a variety of methods, including transmission electron microscopy (TEM), *in vitro* killing assays, and treatment of infected G. mellonella.

The definition of genomic islands (GIs) has been loosened over time to include any chromosomal DNA segment with evidence of horizontal transfer (11). We have introduced the term integrative genetic elements (IGEs) to mean that subset of GIs whose integration into the chromosome can be ascribed to a self-encoded integrase, either from the tyrosine or serine recombinase families (12). The IGE class includes prophages, integrative and conjugative elements (ICEs), and satellites. We have developed two complementary computational tools, Islander (13) and TIGER (12), that identify and precisely map IGEs within bacterial (and archaeal) genome sequences. This software reveals the bacterial host, complete sequence, and precise ends of each prophage. Knowledge of the prophage sequence enables its genome engineering to yield a lysogeny-disabled variant safe for therapy, for example through long amplicon PCR and Gibson assembly, with “rebooting” by introduction and growth in the target bacterium (14). Knowledge of the host enables identification of prophage-laden bacteria that are very close relatives of any given target bacterium, increasing the likelihood that the phages produced will be efficacious on the target. Our powerful computational prophage prediction software facilitates engineering with existing methodologies (3, 14, 15). This set of computational, molecular biology, and microbiology tools together constitute a technology platform for on-demand production of phage therapies against bacterial pathogens (Fig. 1). As an initial demonstration of this approach, we produced five engineered lysogeny-disabled phages that kill Pseudomonas aeruginosa PAO1 in liquid culture as well as in a waxworm model of infection. We foresee application of this platform to develop prepared or on-demand phage collections to target nearly any bacterial pathogen or to control undesirable components of environmental or clinical microbiomes.

## RESULTS

### Identification of prophage-laden close relatives of the target bacterial strain.

Each IGE encodes an integrase that catalyzes recombination between an attachment site in the circular IGE (*attP*) and one in the bacterial chromosome (*attB*), yielding the IGE integrated into the chromosome flanked by left (*attL*) and right (*attR*) attachment sites. We have developed two complementary algorithms that identify IGEs, including prophages. Islander identifies IGEs encoding a tyrosine integrase with *attB* in a tRNA or tmRNA gene (13) (Fig. 1A, right); TIGER identifies IGEs encoding both tyrosine and serine integrases with no bias toward *attB* context (12) (Fig. 1A, left). Both methods are unique relative to competitors in their ability to precisely map *attL* and *attR* for each IGE; moreover, they have superior information retrieval properties. Prior to the publication of TIGER, Bertelli et al. (16) evaluated 20 genomic island (GI)-finding methods with two benchmarking systems: (i) GI-positive and GI-negative segments from 104 bacterial chromosomes and (ii) 80 “gold standard” GIs from six bacterial chromosomes. Islander was top ranked by both systems for precision (in the information retrieval sense), with values of 0.971 for GI-positive or -negative segments and 1.000 for gold standards. Using the benchmarks and evaluation tools of Bertelli et al., we measured the precision of TIGER as 1.000 in both systems (12). Thus, our two methods have better precision, by two measures, than any of the other 19 tested methods. Recall values were lower than for other methods by these benchmarking systems, which we attribute partly to the inclusion of many entries among the GI-positive segments and gold standards that are not associated with integrases and are therefore neither IGEs nor intact prophages. On our own set of 63 gold standard IGEs (not loosely defined GIs) from the same six chromosomes as Bertelli et al. (and one additional chromosome), we measured precision and recall for TIGER at 0.952 and 0.952, respectively, and for Islander at 1.000 and 0.349, respectively (12); the low recall of Islander is expected from its inability to find IGEs that integrate in sites other than tRNA or tmRNA genes (13). Aside from these superior information retrieval properties, TIGER and Islander are also unique in their mapping precision (in the sense of the exactness of the IGE genomic coordinates that they call); both methods identify the identity blocks at the *attL* and *attR* termini of the IGE. A prophage calling tool was developed along with TIGER and benchmarked against a set of temperate phage isolate genomes from NCBI as standards and against bona fide ICEs, mock GIs, and GI-negative chromosomal segments as negatives; it was shown to have 0.982 recall and 0.997 precision (12). With Islander, TIGER, and our prophage caller, we determined the prophage content of the 26 genome-sequenced Pseudomonas aeruginosa strains that were available from the American Type Culture Collection (ATCC), totaling 44 prophages. Two IGE-rich P. aeruginosa strains (referred to here as Pae5 and Pae1505) available from ATCC are close relatives of our target strain PAO1 (see Fig. S1 in the supplemental material). Pae5 has 12 IGEs, including one filamentous prophage and five additional prophages (41Z, 42argF, 44G, 52yheS, and 64L). Strain Pae1505 has 10 IGEs, including one filamentous and two additional prophages (43spxA and 52S) (see Table S1 in the supplemental material). IGE names indicate length (in kilobase pairs) and insertion site gene (a single letter representing the identity of a tRNA gene). Filamentous phages tend not to lyse bacteria and are therefore less suitable for therapy (17). All the prophages in these two strains encode only tyrosine integrases; however, our software is capable of finding prophages with serine integrases as well.

10.1128/mSystems.00659-20.1 FIG S1 Phylogenetic tree and prophage counts of Pseudomonas aeruginosa strains available from ATCC. For the 25 genome-sequenced P. aeruginosa strains available from ATCC in October 2017 (and P. aeruginosa PA14 and as an outgroup to root the tree but not shown, P. pseudoalcaligenes KF707), the nucleotide sequences of the seven genes used for multilocus sequence typing (51) were taken. Sequences were aligned using Muscle separately for each gene, and the alignments were concatenated, yielding a 2,915-bp alignment. A maximum likelihood tree was built using FastTree 2.1.10. Nodes with support values below 0.5 are shown in gray. Strains are designated by ATCC number or other names, with those used in this study in color; the number of prophages determined by Islander/TIGER (12, 13) are given. Download FIG S1, PDF file, 0.07 MB. Copyright © 2020 Mageeney et al. 2020 Mageeney et al. This content is distributed under the terms of the Creative Commons Attribution 4.0 International license.

10.1128/mSystems.00659-20.9 TABLE S1 Integrative genetic element predictions for strains Pae5 and Pae1505. Phages are highlighted in green. Download Table S1, XLSX file, 0.01 MB. Copyright © 2020 Mageeney et al. 2020 Mageeney et al. This content is distributed under the terms of the Creative Commons Attribution 4.0 International license.

### Ensemble validation of prophages.

**FIG 2:**
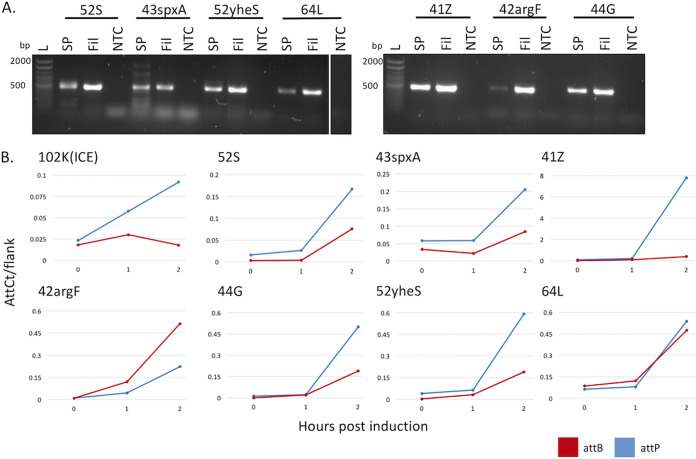
Validation of active prophages. (A) PCR confirmation of soaked clearings from filtrates spotted onto strain PAO1 (SP), filtrate from 2 h MMC induction (Fil), and phage buffer (PB) only control (NTC). Primers are listed in Table S2 in the supplemental material. All phages are released into the filtrate and can infect PAO1, shown by the ∼500-bp band in all SP and Fil lanes. Lane L, Invitrogen 100-bp ladder, 1.2% agarose gel. (B) AttCt (18) analysis of IGEs identified by TIGER and Islander. Normalized *attP* (blue) and *attB* (red) counts are shown. Postexcision replication is indicated by elevated levels of *attP* relative to *attB*.

We sought to determine which of our suspected prophages were able to excise from the bacterial chromosome, produce phage particles, and infect our target strain PAO1. Strains Pae5 and Pae1505 were induced by treatment with mitomycin C; samples were collected at 0, 1, and 2 h, and DNA was prepared from three sample types: a cell pellet, sample from a supernatant filtrate (which should contain free phage particles), and a spot plaque of the filtrate grown on a lawn of PAO1. Precise mapping of the prophages by Islander/TIGER allowed design of PCR tests of the circular junction of each excised prophage; PCR products were not detected for the filamentous phages, but they were detected for the other seven prophages, in all three sample types of the 2-h time points (Fig. 2A). We reexamined all cell pellet DNA samples using deep sequencing, first with our mobilome discovery software Juxtaposer (18) that confirmed the excision products *attP* and *attB*, for six of these seven prophages. We also applied our quantitation tool AttCt (18) to analyze *attP* and *attB* yields (normalized to *attL* and *attR*); higher levels of the former indicate postexcision replication. This analysis reveals (Fig. 2B) interesting biological differences in the onset of excision and replication rates among the prophages, even along this abbreviated time course. One wild-type (WT) phage from each source bacterium, 42argF and 52S, was isolated from each spot plaque by further plaque purification. Although not all prophages are inducible by mitomycin C, the methods for validation are well suited to work with any number of alternative induction strategies, including pH and temperature shifts, UV induction, spontaneous induction, and chemical induction (19–22).

10.1128/mSystems.00659-20.10 TABLE S2 Primers used. Download Table S2, DOCX file, 0.01 MB. Copyright © 2020 Mageeney et al. 2020 Mageeney et al. This content is distributed under the terms of the Creative Commons Attribution 4.0 International license.

### Genomic content of prophages.

Dot plots of the prophage genomes allowed us to reject two prophages (43spxA and 52yheS) as being similar to others, leaving five unique prophages that we proceeded to develop for therapy (Fig. S2; Table 1). Annotation of these prophage genomes (Fig. S3) revealed genes for phage structural proteins (capsid, tail, terminase, and portal) and promoting lysis (endolysin and holin); only 42argF contained genes predicted to encode deleterious proteins (FimA, a known virulence factor [23], and a *hic* toxin-antitoxin system [24]). 42argF also encodes an endosialidase, which allows phage to recognize and degrade bacterial polysaccharide capsules (25), and an anti-CRISPR AcrF6 protein (Fig. S3).

**TABLE 1:** Characterization of phages isolated and engineered from P. aeruginosa strains Pae5 (strain 2192; NCBI accession no. CH482384.1) and Pae1505 (ATCC 27853; NCBI accession no. CP015117.1)^*a*^

Characteristic	41Z	42argF	44G	52yheS	64L	43spxA	52S
Source	Pae5	Pae5	Pae5	Pae5	Pae5	Pae1505	Pae1505
Genome coordinates	6656569− 6697503	3267622− 3309210	6510079− 6553748	1086097− 1137790	828795− 892684	4928948− 4971800	6099420− 6151331
WT recovered	−	+	−	−	−	−	+
Δ*int* mutant constructed	+	+	+	−	+	−	+
Plaque size (Δ*int* vs WT)	N/A	Same	N/A	N/A	N/A	N/A	Same
Tail length (nm)	158.554 ± 0.014 (*Siphoviridae*)	128.778 ± 0.002 (*Siphoviridae*)	118.894 ± 0.009 (*Siphoviridae*)	N/A	148.214 ± 0.004 (*Siphoviridae*)	N/A	77.647 ± 0.006 (*Siphoviridae*)
Capsid type	Icosahedral	Icosahedral	Icosahedral	N/A	Icosahedral	N/A	Prolate
Capsid height × width (nm)	63.253 ± 0.007 × 65.060 ± 0.003	52.907 ± 0.002 × 57.355 ± 0.008	64.159 ± 0.005 × 59.204 ± 0.004	N/A	57.143 ± 0.004 × 54.167 ± 0.002	N/A	62.353 ± 0.003 × 22.941 ± 0.003

a N/A, not available.

10.1128/mSystems.00659-20.2 FIG S2 Genomic dot plot of seven active prophages. The genomes of all seven phages were concatenated into a single file, andGepard software (46) was applied to create a dot plot. Phage names are listed on the axes. The diagonal line has a few gaps that result from gaps in the P. aeruginosa genome sequencing. The red box shows the high level of similarity between 43spxA and 42argF. The green box shows similarity between 52S and 52yheS. Download FIG S2, PDF file, 0.6 MB. Copyright © 2020 Mageeney et al. 2020 Mageeney et al. This content is distributed under the terms of the Creative Commons Attribution 4.0 International license.

10.1128/mSystems.00659-20.3 FIG S3 Genomic maps of the five prophages selected for engineering. Genes are marked above the line for forward orientation and below the line for reverse orientation. Gene functions were predicted using HHpred (44, 45) searching the Pfam-A and PDB protein databases. Predicted functional categories were lysogeny genes (yellow), lytic genes (red), structural genes (green), functional genes in other categories (blue), and hypothetical genes (grey). Abbreviations: Y-int, tyrosine integrase; HTTC, head-to-tail connector protein; ssb, single-stranded DNA binding protein. The pink boxes represent genome segments that were deleted during Δ*int* phage production. The scale bar represents 5 kbp. Download FIG S3, PDF file, 0.2 MB. Copyright © 2020 Mageeney et al. 2020 Mageeney et al. This content is distributed under the terms of the Creative Commons Attribution 4.0 International license.

### Engineering of Δ*int* lysogeny-disabled prophages.

Conversion of a temperate phage into a lytic phage requires deletion of at least one key lysogeny determinant. Others have successfully targeted the repressor gene (3) or the “lysogeny control region” (integration and prophage maintenance gene cluster) (14) for this purpose. For high-throughput genome engineering of phages with diverse bacterial hosts, the repressor gene may be difficult to identify reliably amid other helix-turn-helix proteins. We chose to delete the integrase gene (*int*), which is essential for lysogeny and readily identifiable. Conveniently for deletion, *int* is typically located at one end of the prophage genome (12). The integrase gene and its surrounding regions, including the *attL* and *attR* sites, were deleted by using PCR primers to generate long, partially overlapping amplicons comprising all included prophage sequences, followed by Gibson assembly of the amplicons and transformation into the target host (Fig. 1D) (14). Each amplicon was 8 to 15 kbp in length, producing terminal overlaps with neighboring amplicons of 40 to 60 bp (Table S2). Gibson assembly was performed with the three to five partially overlapping amplicons, and the products were transformed into electrocompetent (41Z, 42argF, 44G, and 52S) or chemically competent (64L) PAO1. The transformed bacteria were plated, and phages were recovered from plaques at 16 h postplating. PCR was used to verify Δ*int* junctions in the phage genomes (Fig. S4).

10.1128/mSystems.00659-20.4 FIG S4 Confirmation of phages. PCR tests (Table S2) were designed for the *attP* formed by excision of each WT phage and for the Δ*int* deletion of each engineered phage. L, Invitrogen 100-bp ladder, 1.2% agarose gel. Download FIG S4, PDF file, 0.3 MB. Copyright © 2020 Mageeney et al. 2020 Mageeney et al. This content is distributed under the terms of the Creative Commons Attribution 4.0 International license.

### Phage morphology.

Transmission electron microscopy (TEM) was used to determine morphology for each phage (Fig. S5). All five phages had long, flexible, noncontractile tails characteristic of *Siphoviridae*. Four of the phages had icosahedral capsids, whereas 52S had a prolate head. Head and tail dimensions were measured from TEM images (Table 1). Four of the five phages measured 200 to 300 nm in length, whereas 52S (both WT and Δ*int*) measured 150 nm in length.

10.1128/mSystems.00659-20.5 FIG S5 Electron microscopy (EM) of phages. EM grids were prepared with >10^9^ PFU/ml of fresh lysate, stained with uranyl acetate alternative stain (Ted Pella, catalog no. 19485, using gadolinium acetate tetrahydrate), and imaged with a Themis Z transmission electron microscope operated in HAADF-STEM mode (image contrast inverted for clarity). Scale bars representing 100 nm are shown. Download FIG S5, PDF file, 0.4 MB. Copyright © 2020 Mageeney et al. 2020 Mageeney et al. This content is distributed under the terms of the Creative Commons Attribution 4.0 International license.

### Engineered phages kill strain PAO1 in liquid culture.

**FIG 3:**
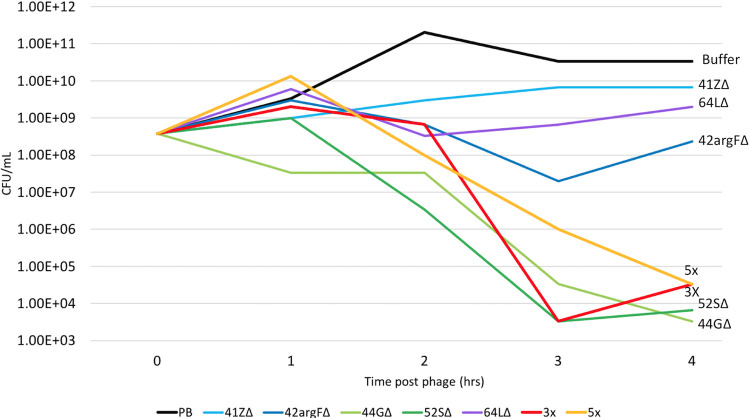
Engineered phages kill strain PAO1 in liquid culture. PAO1 liquid cultures were grown to mid-log phase (OD_600_ of 0.4 to 0.6) and then infected with phage at an MOI of 10 for each single phage (blue lines), two phage cocktails (red and orange lines) (3X phage cocktail is 44GΔ, 52SΔ, and 64LΔ, and 5X phage cocktail is 3X plus 41ZΔ and 42argFΔ), or a buffer-only control (black line). Bacterial cells were plated every hour for 4 h, and CFU were calculated. PAO1 with buffer has increased growth over the time course, while all phage samples show decreased growth over the time course. Decreased growth indicates that the phages are killing PAO1. Replicate number 1 is shown; other replicates are shown in Fig. S6.

The Δ*int* phages were tested for the ability to kill the target pathogen PAO1 in liquid culture. Each phage stock was added to a mid-log-phase PAO1 culture at a multiplicity of infection (MOI) of 10, and the culture was incubated at 37°C with shaking (250 rpm) for 24 h. Aliquots were removed from the culture at 1-h intervals for the first 4 h following phage addition in order to assess the degree and timing of bacterial death and phage proliferation in the culture. By 4 h following phage addition, all cultures exposed to Δ*int* phage showed reduced bacterial titers relative to mock-exposed cultures (i.e., those receiving buffer only), indicating that each of the Δ*int* phages is capable of killing PAO1 (Fig. 3 and Fig. S6). Additionally, phage titers increased at least 10-fold, and as much as 1,000-fold, in all of the cultures by 4 h (Fig. S7).

10.1128/mSystems.00659-20.6 FIG S6 Engineered phages kill strain PAO1 in liquid culture. Two additional independent replicates of the experiment in Fig. 3 are shown. Download FIG S6, PDF file, 0.2 MB. Copyright © 2020 Mageeney et al. 2020 Mageeney et al. This content is distributed under the terms of the Creative Commons Attribution 4.0 International license.

**FIG 4:**
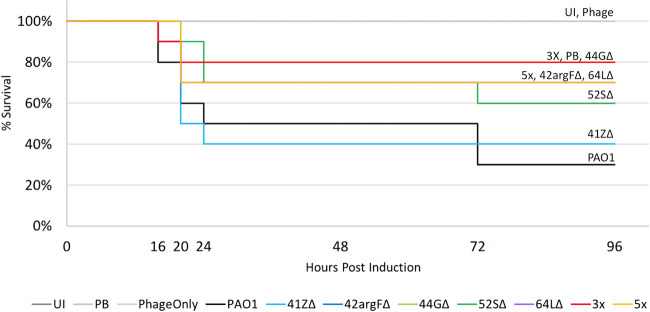
Engineered phages protect Galleria mellonella larvae from bacterial infection. Cohorts of 10 G. mellonella larvae were injected with 10^3^ CFU/ml of strain PAO1 in the second to last right proleg. After 30 min, each larva was injected with an MOI of 10 of either a single phage (blue line), a phage cocktail (red and orange lines) (3X cocktail is 44GΔ, 52SΔ, and 64LΔ, and 5X cocktail is 3X cocktail plus 41ZΔ and 42argFΔ), or PB (black line). Three additional controls (gray lines) were used: uninjected (UI), PB for both injections, and PB followed by the 5X phage cocktail (Phage). Increased survival indicates that the phages are killing the PAO1 *in vivo*. Replicate one is shown; replicates two and three are shown in Fig. S7.

10.1128/mSystems.00659-20.7 FIG S7 Engineered phages protect Galleria mellonella larvae from bacterial infection. Two additional independent replicates of the experiment in Fig. 4 are shown. Download FIG S7, PDF file, 0.08 MB. Copyright © 2020 Mageeney et al. 2020 Mageeney et al. This content is distributed under the terms of the Creative Commons Attribution 4.0 International license.

Two mixtures (cocktails) of Δ*int* phage stocks were designed: cocktail 3X, comprised of phages 44GΔ, 52SΔ, and 64LΔ, and cocktail 5X, comprised of the 3X phages plus 41ZΔ and 42argFΔ. These cocktails were added to PAO1 cultures at an MOI of 10 (MOI of 3.3 for each Δ*int* phage in 3X, and MOI of 2 for each in 5X), aliquots were removed from the cultures at 1-h intervals over the course of 4 h, and the bacterial and phage titers within each aliquot were measured as described above. Both cocktails reduced bacterial titers by 10^6^-fold within 4 h postexposure.

### Δ*int* phage therapy protects waxworms from PAO1 infection.

We sought to determine whether these phages confer therapeutic effects in the context of a PAO1 infection model. The Galleria mellonella larva (waxworm) model is convenient, low cost, and well established for *Pseudomonas* and other bacterial infections and testing of antimicrobial therapies (26–30). Importantly, antimicrobial therapies, including phage therapy, that are efficacious in waxworm models of infection generally also show efficacy in mammalian models of infection (29, 31).

Each larva was injected with 50 CFU of strain PAO1, and 30 min later injected with buffer (negative control), a single Δ*int* phage (MOI of 10), or a phage cocktail (MOI of 10 for sum of phages in cocktail), with each therapy tested in 10 larvae (Fig. 4). This experiment was replicated twice more (Fig. S7). Waxworms treated with buffer alone showed only 30 to 40% survival by 3 days postexposure (black line, Fig. 4 and Fig. S7); mortality was due to PAO1 rather than the injection procedure in that mock-infected larvae (injected with buffer instead of PAO1 and then injected a second time to simulate treatment) showed 100% survival (gray line). In contrast, larvae treated with single Δ*int* phage showed 40% to 80% survival. Larvae treated with phage cocktails showed further gains in survival, ranging from 70% to 90% (red and orange lines). Treatment with phage in the absence of infection had no deleterious effect on survival (gray line). These results indicate that Δ*int* phages, particularly when combined as a cocktail, can protect waxworms against PAO1 infection.

## DISCUSSION

This report validates a new technology platform that enables on-demand production of therapeutic phages against a bacterial pathogen of interest. The first step in our approach is to use two complementary computational tools (Islander and TIGER) to identify and precisely map the prophages present in genome sequences of bacteria closely related to the pathogen. We have carried this out for many genomes (12), and we plan to extend our analysis to all available genome sequences. Preemptive creation of prophage databases in this manner allows for rapid turnaround in phage therapy cases where time is limited—the physician can use the pathogen’s genome sequence, 16S rRNA gene sequence, or multilocus sequence typing (MLST) sequences to place it on a phylogenetic tree, to identify close relatives bearing large numbers of prophages. These prophages then serve as starting material for construction of synthetic phages that are engineered for therapeutic use through deletion of genes (such as *int*) essential for lysogeny. Identification of numerous prophages from close relatives of bacterial targets has the potential to overcome many of the hurdles associated with environmental phage isolation, including defining optimal hosts, sequencing isolated phages, and annotation of deleterious gene products. Once prophages are identified for the host of interest, phage engineering can be completed by previously established methods (3, 14, 15).

In principle, our approach is pathogen-agnostic and could be extended to any pathogen group. Raw numbers of prophages are not generally a problem; Fig. S8 in the supplemental material shows the average prophage count per genome for diverse ESKAPE (Enterococcus faecium, Staphylococcus aureus, Klebsiella pneumoniae, Acinetobacter baumannii, Pseudomonas aeruginosa, and *Enterobacter* species) and tier 1 select agent pathogen species. Ten pathogen species have double or more the average prophage count of all bacteria, although two (Burkholderia mallei and Francisella tularensis) do not. However, particular bacterial groups may present practical problems. Our study host, P. aeruginosa PAO1, has well-defined transformation protocols, which are not available for all pathogens (32–34), but circumventions are available (14). Many bacterial systems have phages with narrow host ranges (35, 36); host range factors are still being investigated but have been linked to tail fiber mutations or host receptor mutations (36–38). While we did not encounter this problem, the validation methods described allow for prescreening of prophages that are inactive against the target pathogen prior to engineering. Further, Gibson assembly allows for additional engineering at other sites in the phage genome aimed at expanding its host range. Once such challenges are solved, both the computational and laboratory methods described will be suitable for high-throughput scale-up and can be further streamlined to support a phage factory that prepares therapeutic phage sets for any and all pathogens.

10.1128/mSystems.00659-20.8 FIG S8 Prophages in pathogens. The Islander/TIGER prophages for 2,031 bacterial genomes were taken from reference 12, identifying an average of 1.07 prophages per genome. Averages are also shown for ESKAPE pathogens and tier 1 select agents (numbers of genomes tested in parentheses). Download FIG S8, PDF file, 0.1 MB. Copyright © 2020 Mageeney et al. 2020 Mageeney et al. This content is distributed under the terms of the Creative Commons Attribution 4.0 International license.

## MATERIALS AND METHODS

### Prophage detection.

Two IGE discovery algorithms, Islander (13) and TIGER (12), were applied to 2,023 *Pseudomonas* genomes downloaded from GenBank in October 2017. Predicted prophage genomes were annotated. The multiPhATE pipeline (39), which calls Glimmer (40), Prodigal (41), and Phanotate (42), was used to predict open reading frames. Prokka (43) tFind, and rfind (13) were used for functional annotation. HHPRED (44, 45) was used to further characterize phage functions to compare homology of each protein annotated against the Pfam-A v32.0 and PDB_mmCIF70_28_Nov databases. Prophage genomes were compared to each other to identify similar prophages. Gepard (46) was used to generate dot plots for the seven nonfilamentous prophage sequences. Phage genome maps were created using Easyfig (47).

### Prophage induction.

Prophage-laden strains P. aeruginosa 2192 (ATCC 39324; Pae5) and P. aeruginosa Boston 41501 (ATCC 27853; Pae1505) were purchased from ATCC. Strains were grown overnight in LB broth, diluted 1:100, and grown to an optical density at 600 nm (OD_600_) of 0.4 to 0.5. Samples (1 ml) were collected at 0, 1, and 2 h after treatment with 1 μg/ml mitomycin C. Cells were pelleted at 16,000 × *g* for 2 min; supernatant was removed and filtered through a 0.22-μm filter. Genomic DNA was isolated using the Qiagen DNeasy blood and tissue kit (Qiagen, catalog no. 69504). Filtrates were spotted onto a lawn of P. aeruginosa PAO1 (a kind gift from Annette LaBauve) with 0.5% LB soft agar and incubated overnight. Top agar was collected from the cleared spot regions and soaked in 0.5 ml phage buffer (PB) (100 mM NaCl, 8 mM MgSO_4_·7H_2_O, 25 mM Tris-HCl [pH 7.4]) overnight at 4°C and filtered through a 0.22-μm filter.

### Monitoring prophage activity through deep sequencing.

P. aeruginosa (Pae5 and Pae1505) genomic DNA sequencing libraries were prepared using Nextera DNA library prep kit (Illumina, catalog no. FC-121-1031) and utilizing the Nextera DNA Sample Preparation Index kit (Illumina, catalog no. FC-121-1011) following the manufacturer’s recommended protocol. DNA samples and libraries were quantified using Qubit high-sensitivity DNA assay kit (Thermo Fisher Scientific, catalog no. Q32854). Libraries were pooled in equal quantity and combined to multiplex to make a final library, and quality control (QC) was done again using Qubit quantification kit and Agilent bioanalyzer using high sensitivity DNA chip (Agilent Biotechnology, catalog no. 5067-4626). The final combined library was sequenced using Illumina technology on a NextSeq 500 sequencer using high-output 300-bp single-end read sequencing kit.

Sequencing reads were processed through BBDuk for quality filtering. The filtered reads were analyzed with Juxtaposer (18) to find mobile elements in the bacterial genomes. Briefly, Juxtaposer searches for recombinant reads relative to the reference genome sequence, which includes reads for the *attP* and *attB* products of IGE excision. Finally, attCt software (18) was used to determine counts for each IGE of *attL*, *attR*, *attB*, and *attP*, reporting *attP*/F and *attB*/F values, where F = *attL* + *attR *+ 2 *attB*).

### Wild-type phage isolation.

Soaked filtrates from mitomycin C (MMC) induction were serially diluted in SM phage buffer. One hundred microliters of P. aeruginosa PAO1 was infected with 100 μl of each phage dilution, allowed to adsorb 20 min, and plated using 4.5 ml of 0.5% LB soft agar. Plaques were isolated in SM phage buffer, and PCR tests for the *attP* site were performed to determine which plaque was each phage (primers listed in Table S2 in the supplemental material).

### Gibson assembly of Δ*int* phages.

Primers were designed to obtain long PCR fragments with overlapping joints suitable for Gibson assembly strategies (Table S2). Primers for the integrase deletion were created with artificial 40-bp overlaps. Briefly, a 20-bp primer was created around the integrase gene, and a 20-bp flanking region was added to the 5′ end of the primer from the opposing end of the Δ*int* circular junction. Long PCR was performed using the NEB Phusion High-Fidelity DNA polymerase master mix. PCR conditions were as follows: (i) 98°C for 2 min; (ii) 35 cycles with 1 cycle consisting of 98°C for 30 s, melting temperature (*T_m_*) minus two degrees for 30 s, and 72°C for 1 min/kbp; and (iii) 72°C for 10 min. NEB Gibson Assembly master mix was used to ligate phage fragments together. Then, 0.3 pmol of each long PCR product was incubated at 50°C for 15 min for three-fragment phages (41ZΔ, 42argFΔ, and 44GΔ) and 60 min for four or more fragment phages (52SΔ and 64LΔ).

### Competent cell preparation and transformation.

Electrocompetent PAO1 cells were prepared as previously described (48). Briefly, an overnight culture of strain PAO1 was diluted 1:100 and grown to an OD_600_ of 0.5. Cells were pelleted 2 min at 16,000 × *g*, and supernatant was removed. Cells were washed twice with 300 mM sucrose and resuspended in 300 mM sucrose. For all phages except 64L, 5 μl of the undiluted or 1:3 diluted Gibson assembly reaction was delivered into electrocompetent cells using a Bio-Rad electroporator (2.5 kV, 200 Ω, 25 μF, 2 mm). Cells were allowed to recover in 1 ml of LB medium for 1 h, shaking at 37°C. Cells were pelleted for 2 min at 16,000 × *g*, 800 μl of medium was removed, and cells were resuspended in the remaining medium. Transformations were diluted in SM phage buffer by 10-fold and 1,000-fold. Two hundred microliters of the transformed cells or dilutions was used to infect 100 μl of mid-log PAO, incubating for 20 min, plating with 0.5% LB soft agar, and incubating overnight at 37°C.

Phage 64L did not produce plaques using electrotransformation protocols. Chemically competent (CC) PAO1 was prepared as previously described (49). Overnight cultures were diluted 1:100 and grown to an OD_600_ of 0.8. Cells were chilled and harvested by centrifugation, washed with 100 mM MgCl_2_, and incubated on ice in 175 mM CaCl_2_ for 20 min. The final cell pellet was resuspended in 100 mM CaCl_2_. Fresh competent cells (200 μl) were incubated with various amounts of Gibson assembly product DNA for 60 min on ice, heat shocked at 42°C for 1 min, chilled, and recovered in 1 ml of LB broth for 1 h prior to plating with 100 μl of mid-log PAO1 and 4 ml of 0.5% LB top agar (TA).

### Recovery of Δ*int* mutant phages.

Plaques recovered from the transformations were picked, put in 0.1 ml SM phage buffer, and confirmed through PCR with primers designed in the flanking region of the deletion (Table S2). Phage stocks were prepared by titrating on plates to obtain a web pattern of lysis. Plates were flooded with 8 ml of SM phage buffer overnight at 4°C, filtered through a 0.22-μm filter. Phage stocks were stored in 0.3% sucrose.

### Electron microscopy.

Electron microscopy grids were prepared with fresh lysates at >10^9^ PFU/ml. Ten microliters of lysate was added to a carbon grid (Ted Pella, catalog no. 1813) and allowed to incubate 10 min. The grids were washed twice with 10 μl of water for 2 min. Grids were stained with 10 μl of uranyl acetate alternative stain (Ted Pella, catalog no. 19485, using gadolinium acetate tetrahydrate) for 2 min and wicked off. Grids were allowed to dry at room temperature in a chemical fume hood for 1 h and stored in a grid box until imaging.

Transmission electron microscopy observations were conducted using a Themis Z transmission electron microscope (Thermo Fisher Scientific, Hillsboro, OR, USA) operated in scanning transmission electron microscopy (STEM) mode at an accelerating voltage of 300 kV. Image signals were collected using a high angle annular dark field (HAADF) detector, which is sensitive to the heavy elements (Gd) employed in the stain. In the micrographs presented here, the dark-field image contrast has been inverted, with the heavy metal stain showing as dark regions on the images.

Immediately prior to observation, specimens were plasma cleaned (Mobile Cubic Asher, IBSS Group, Burlingame, CA, USA) to reduce the build-up contamination under exposure to the focused electron beam. Plasma cleaning was conducted for 5 min using ambient air at a base pressure of 10^−4 ^torr and power setting of 34 W.

### Liquid killing assays.

Overnight PAO1 cultures were diluted 1:100 to an OD_600_ of 0.02 to 0.04 in LB broth. Cultures were grown to an OD_600_ of 0.4 to 0.5. Cells were pelleted by centrifugation at 3,000 rpm for 5 min. Bacterial pellets were resuspended in 1 ml PB containing a single phage stock at a multiplicity of infection (MOI) of 10 or a phage cocktail with a total MOI of 10; 5 ml of LB broth was added to each culture. Cultures were grown for 24 h, removing 0.5-ml samples at 0, 1, 2, 3, and 4 h after phage addition. Each sample was processed for PFU, CFU, and OD_600_ measurements.

Bacterial cell counts were performed by serially diluting the sample in LB broth to 10^−8^ and spotting 3 μl of each dilution onto a solid LB agar plate and incubating at 37°C for 16 h. PFU were determined by centrifugation of each sample at 3,000 rpm for 5 min, removing 100 μl of supernatant, serial diluting the supernatant to 10^−10^, followed by spotting 3 μl of each dilution onto a 0.5% soft agar overlay plate. Bacterial and phage titers were calculated by counting the number of colonies or plaques, respectively.

### Waxworm phage therapy.

Galleria mellonella larvae were purchased from Timberline (Marion, IL) and stored at 15°C until use within 2 weeks. Only larvae weighing between 0.25 and 0.35 g were used for injection experiments. Larvae were equilibrated at room temperature for 4 h prior to injection. Five microliters of strain PAO1 (10^3^ CFU/ml) was injected into the second to last right proleg using a 250-μl, model 1725 LT syringe (Hamilton, catalog no. 81101) and PB600 repeating dispenser (Hamilton, catalog no. 83700). Thirty minutes after the PAO1 injection, 5 μl phage (MOI of 10) (10^4^ PFU/ml) was injected into the second to last left proleg, and the larvae were incubated at 37°C for 72 h. Death was assessed by melanization and lack of movement at 16, 20, 24, 48, and 72 h postinjection. Kaplan-Meier survival curves were calculated as described previously (50).
