# Supplementary figures and images for: Computational Basis for On-Demand Production of Diversified Therapeutic Phage Cocktails
Source: mSystems. 2020 Aug 11;5(4):e00659-20. doi: 10.1128/mSystems.00659-20 (PMC7426155; doi:10.1128/mSystems.00659-20)

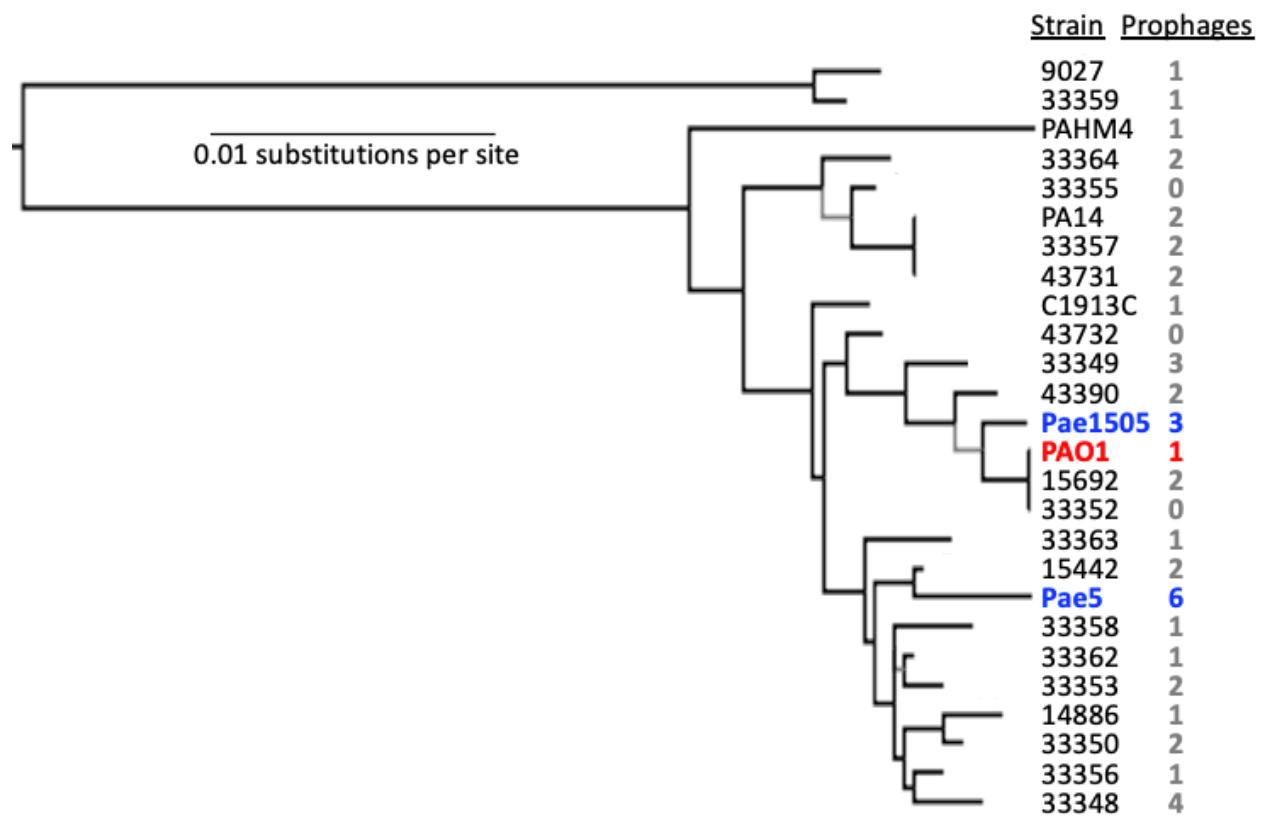

Supplement: FIG S1 [file mSystems.00659-20-sf001.pdf]

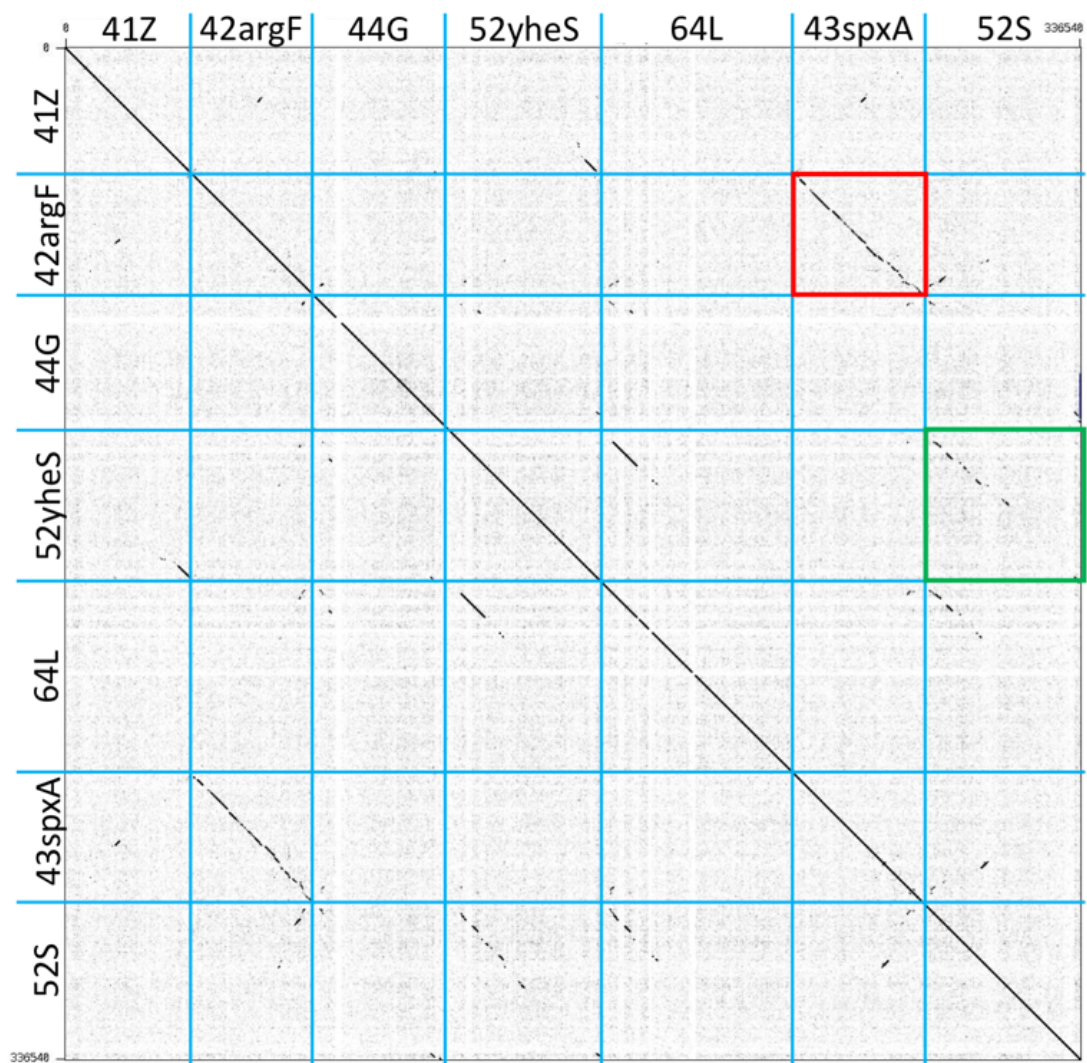

Supplement: FIG S2 [file mSystems.00659-20-sf002.pdf]

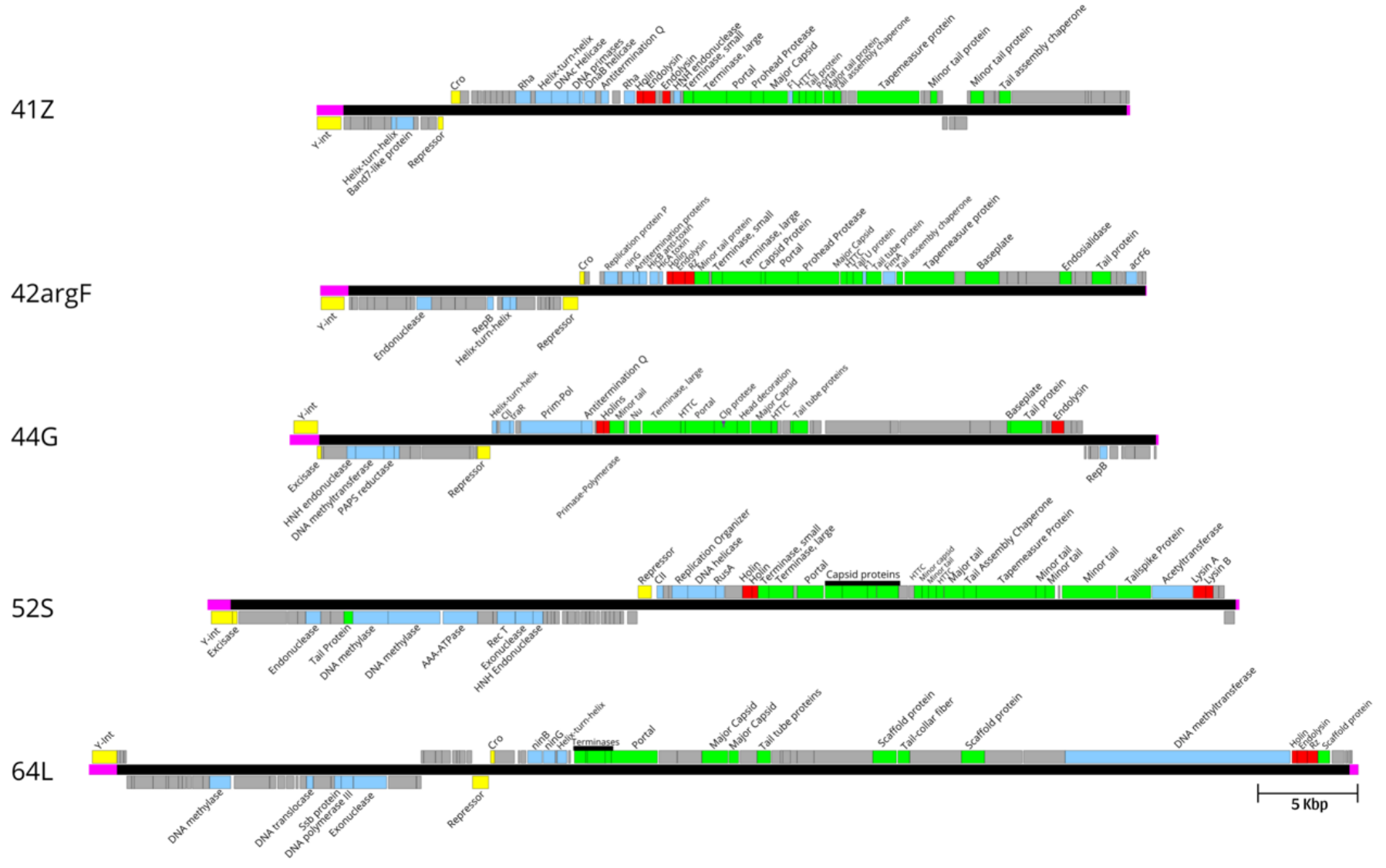

Supplement: FIG S3 [file mSystems.00659-20-sf003.pdf]

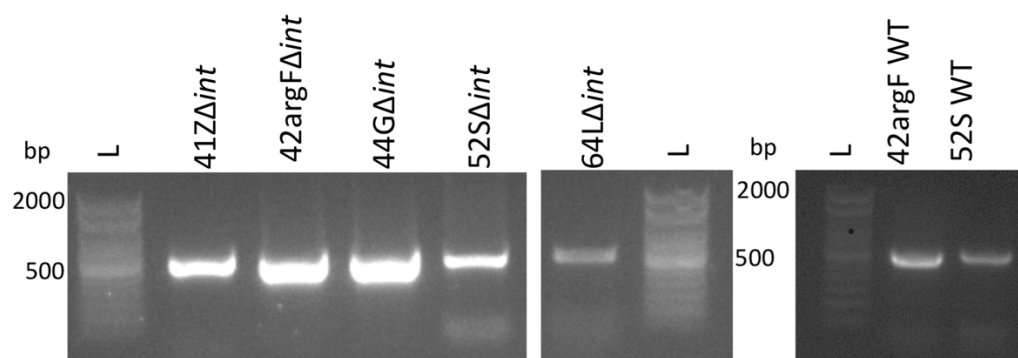

Supplement: FIG S4 [file mSystems.00659-20-sf004.pdf]

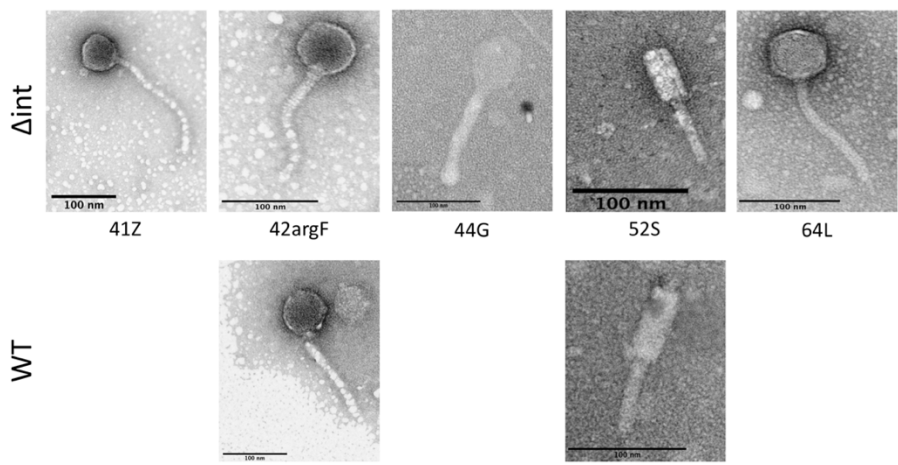

Supplement: FIG S5 [file mSystems.00659-20-sf005.pdf]

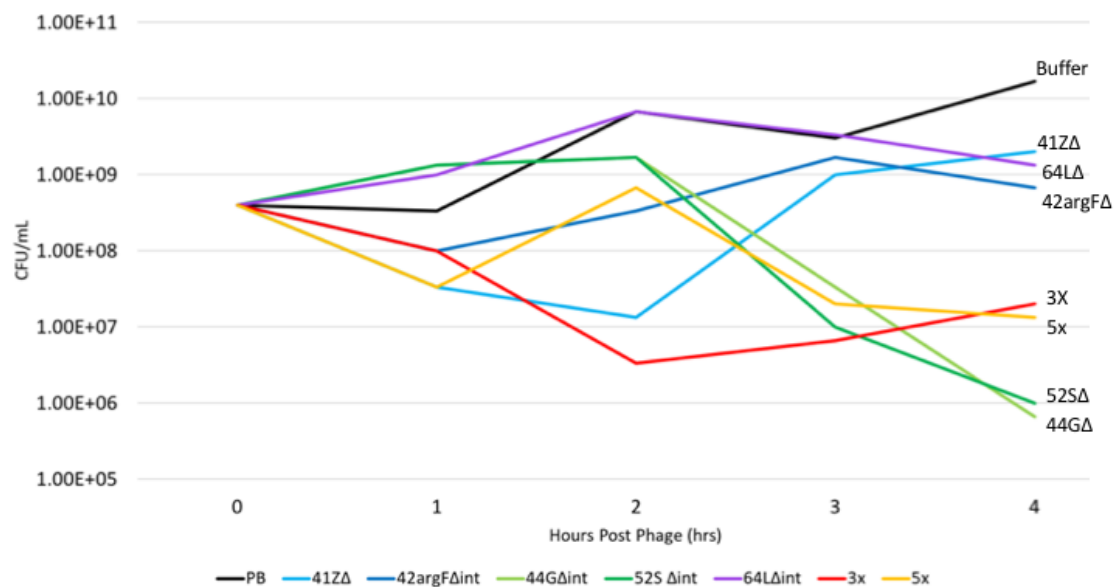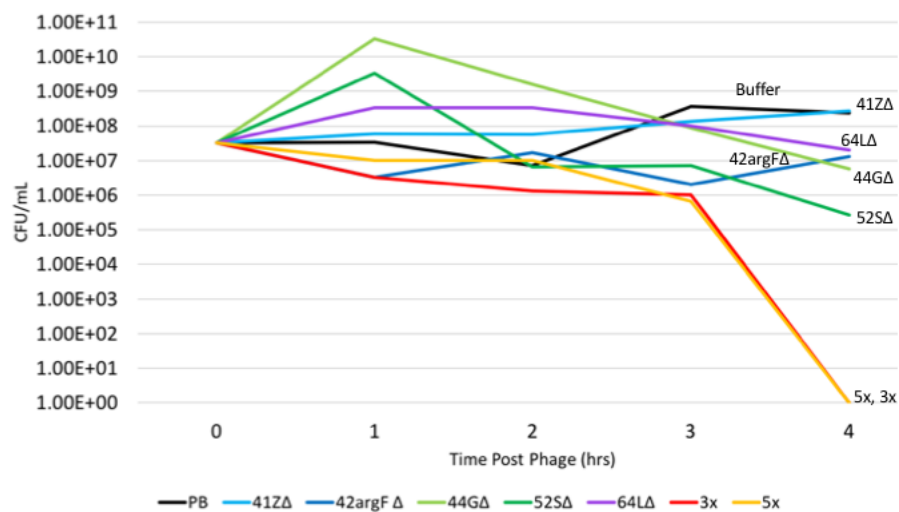

Supplement: FIG S6 [file mSystems.00659-20-sf006.pdf]

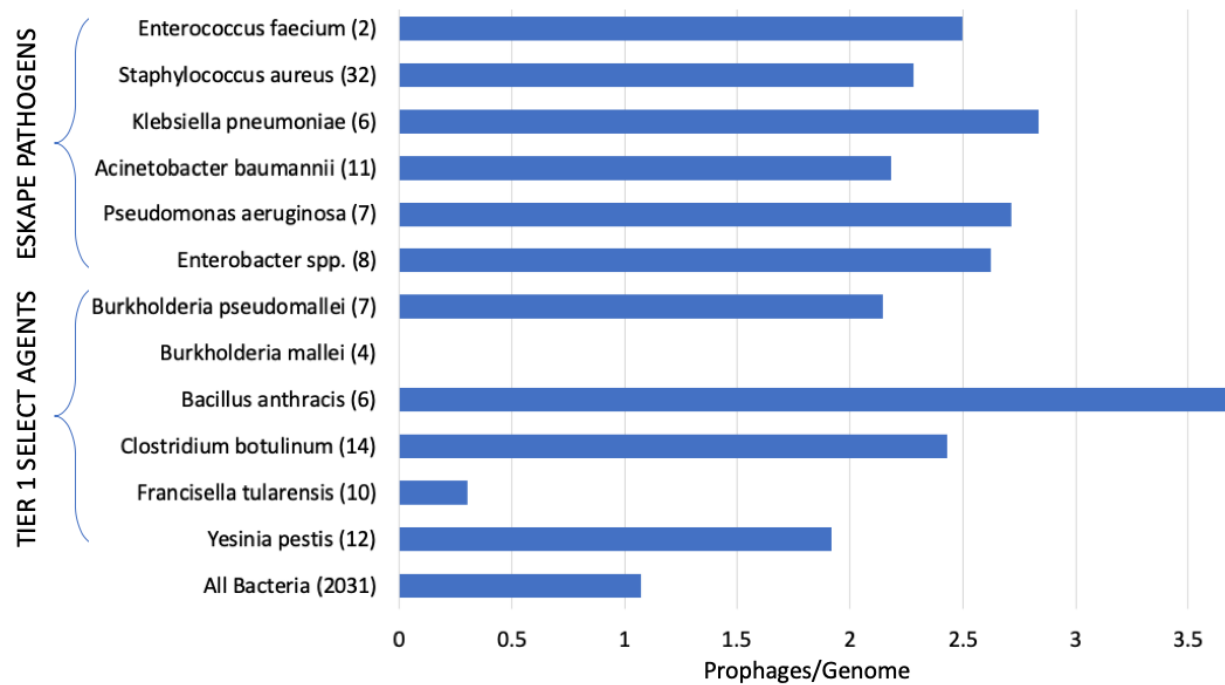

Supplement: FIG S8 [file mSystems.00659-20-sf008.pdf]
