# Supplementary material for: Computational Basis for On-Demand Production of Diversified Therapeutic Phage Cocktails
Source: mSystems. 2020 Aug 11;5(4):e00659-20. doi: 10.1128/mSystems.00659-20 (PMC7426155; doi:10.1128/mSystems.00659-20)
Supplement: TABLE S2 [file mSystems.00659-20-st002.docx]

| **Primer Name** | **Sequence (5’ -> 3’)** | **Use** |
| --- | --- | --- |
| Pae5.52yheS_L | cacgccgcgctggatgct | Detection of attP of excised 52yheS WT phage |
| Pae5.52yheS_R | acggctcggagagcagct |  |
| Pae5.42argF_L | gtccgacagcacaacgag | Detection of attP of excised 42argF WT phage |
| Pae5.42argF_R | cggcaatcctgcctgccg |  |
| Pae5.64L_L | cctatctctatttccgcc | Detection of attP of excised 64L WT phage |
| Pae5.64L_R | aaagcggaagtgccagct |  |
| Pae5.41Z_L | aaacactgtgggtttacg | Detection of attP of excised 41Z WT phage |
| Pae5.41Z_R | cgaggcgacacgatttcg |  |
| Pae1505.43spxA_L | cataagcgccgcgttcgc | Detection of attP of excised 43spxA WT phage |
| Pae1505.43spxA_R | cgcggccatccgtccggc |  |
| Pae1505.52S_L | gcatcgcggcgagccatt | Detection of attP of excised 52S WT phage |
| Pae1505.52S_R | atccgcccgaaagcagcc |  |
| 141.44G.R | CGCAGTCTTGAATTCGGGCG | Detection of attP of excised 44G WT phage |
| 142.44G.L | GCCGTTCTCCCGCACCTTTA |  |
| 65.52SL.dx | AGCACGCCGATGGACAGAT | Detection of deletion in 52S ∆int phage |
| 67.52SR.dx | GGCGGAGGTATGTTATCCCG |  |
| 88.Pae5.41Z.dxL | GTCGAAGGGCGGCAAGAAAG | Detection of deletion in 41Z ∆int phage |
| 89.Pae5.41Z.dxR | GCGACTACACAACCGTCTCA |  |
| 96.Pae5.42argF.dxL | TCGGCAGATAGGCAGTTCCG | Detection of deletion in 42argF ∆int phage |
| 97.Pae5.42argF.dxR | AGTGTGAGCCAGACGTGCTT |  |
| 157.44G.dxL | CTCAGGGAGGGCCACGCGAT | Detection of deletion in 44G ∆int phage |
| 158.44G.dxR | GCTGCGAGATGAGTCGCGTG |  |
| 106.Pae5.64L.dxF | GCAATACGCAACAGCACCAA | Detection of deletion in 64L ∆int phage |
| 107.Pae5.64L.dxR | GACAGGGAAACCGGCTGTTA |  |
| 52S.Af | gcggcatccagagaatgagaaatcatccggggtgggagat | Fragment A of 52S ∆int phage; product length 12463bp; 42bp overlap with fragment B |
| 52S.Ar | gaggaatcccgcgagtggaa |  |
| 52S.Bf | gcttcttgacgacgcggtaa | Fragment B of 52S ∆int phage; product length 12574bp; 41bp overlap with fragment C |
| 52S.Br | ctacgcccgttggtgtcctt |  |
| 52S.Cf | caacgagacgcaccccga | Fragment C of 52S ∆int phage; product length 12619bp; 38bp overlap with fragment D |
| 52S.Cr | tgatccaatgaacggtcagca |  |
| 52S.Df | tgtaaacggcacgaatgctg | Fragment D of 52S ∆int phage; product length 13121bp; 40bp overlap with fragment A |
| 52S.Dr | atctcccaccccggatgatttctcattctctggatgccgc |  |
| 41Z.Af | TGATCTGCCGAGGTGAAAGCCGCGTCTTCGGTGTAGCCAGA | Fragment A of 41Z ∆int phage; product length 14325bp; 39bp overlap with fragment B |
| 41Z.Ar | GCGGAGACGGAATGCCTTTG |  |
| 41Z.Bf | TATCCGTCGCATGGCCTGTT | Fragment B of 41Z ∆int phage; product length 11936bp;48bp overlap with fragment C |
| 41Z.Br | AGTGCCCTCCAAGGATGACC |  |
| 41Z.Cf | TGCAGTGTATTCCGTCGCTCA | Fragment C of 41Z ∆int phage; product length 13279bp; 41bp overlap with fragment A |
| 41Z.Cr | TCTGGCTACACCGAAGACGCGGCTTTCACCTCGGCAGATCA |  |
| 42argF.Af | gcaatggggctgctcgttcATATAACCCCGCACAACCCC | Fragment A of 42argF ∆int phage; product length 13515bp; 39bp overlap with fragment B |
| 42argF.Ar | CCTACAAGTCTGCCACCGTC |  |
| 42argF.Bf | GTCAGCGCCTCGATCACATC | Fragment B of 42argF ∆int phage; product length 13025bp; 45bp overlap with fragment C |
| 42argF.Br | TGTGGAGCGTACTCAACGAC |  |
| 42argF.Cf | GGCTGGGTATCGCTAACCATTA | Fragment C of 42argF ∆int phage; product length 13559bp; 39bp overlap with fragment A |
| 42argF.Cr | ggggttgtgcggggttatatGAACGAGCAGCCCCATTGC |  |
| 44G.Af | GAGCGGGCGGAGGGAATCGACCCACGCGGCCGGGAGAGAG | Fragment A of 44G ∆int phage; product length 14317bp; 47bp overlap with fragment B |
| 44G.Ar | ACCTCGCCGGAGCAACCACA |  |
| 44G.Bf | GCGGCGCTGCTGGTGGTGAC | Fragment B of 44G ∆int phage; product length 13561bp; 42bp overlap with fragment C |
| 44G.Br | TGCGCTGGTCCGAGAGCGAT |  |
| 44G.Cf | GAGTCCTCGGACAAGGCGGCCAA | Fragment C of 44G ∆int phage; product length 14426bp; 40bp overlap with fragment A |
| 44G.Cr | CTCTCTCCCGGCCGCGTGGGTCGATTCCCTCCGCCCGCTC |  |
| 64L.Af | GGATGGCGCATTTGAAGGCATCACTCCGCGGCCTCTTCTTC | Fragment A of 64L ∆int phage; product length 13672bp; 49bp overlap with fragment B |
| 64L.Ar | GGTCACCGTAGGAGCATCCG |  |
| 64L.Bf | CGAGCCTTGGACTACGTGTG | Fragment B of 64L ∆int phage product length 15858bp; 47bp overlap with fragment C |
| 64L.Br | GGTGGTTTTGCTTGCGGTCA |  |
| 64L.Cf | AACCGGAACATGTAAGGGAGC | Fragment C of 64L ∆int phage; product length 15751bp; 41bp overlap with fragment D1 |
| 64L.Cr | CTGCAGGGTGGCCTTGTAGG |  |
| 64L.D1f | AAGTTCTTGGCGGACGTGGA | Fragment D1 of 64L ∆int phage; product length 7999bp; 47bp overlap with fragment D2 |
| 64L_D1r | GCGGCTGACGGACAGAAGAT |  |
| 64L_D2f | GAGGCCTACACGGACTGGG | Fragment D2 of 64L ∆int phage; product length 8907bp; 41bp overlap with fragment A |
| 64L.D2r | GAAGAAGAGGCCGCGGAGTGATGCCTTCAAATGCGCCATCC |  |
