# Supplementary material for: Computational Basis for On-Demand Production of Diversified Therapeutic Phage Cocktails
Source: mSystems. 2020 Aug 11;5(4):e00659-20. doi: 10.1128/mSystems.00659-20 (PMC7426155; doi:10.1128/mSystems.00659-20)
Supplement: FIG S7 [file mSystems.00659-20-sf007.pdf]

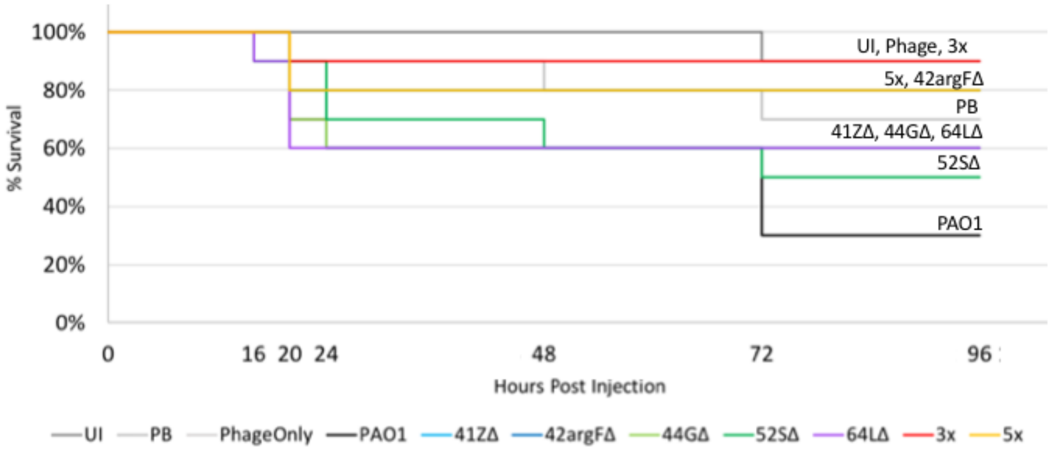
100%
80%
60%
40%
20%
0%
% Survival
0
16
20
24
48
72
96
Hours Post Injection
UI, Phage, 3x
5x, 42argFΔ
PB
41ZΔ, 44GΔ, 64LΔ
52SΔ
PAO1
UI
PB
PhageOnly
PAO1
41ZΔ
42argFΔ
44GΔ
52SΔ
64LΔ
3x
5x

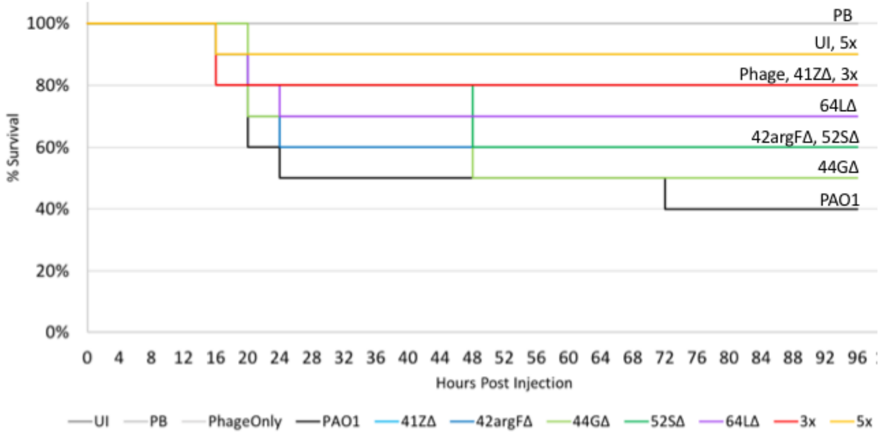
100%
80%
60%
40%
20%
0%
% Survival
0 4 8 12 16 20 24 28 32 36 40 44 48 52 56 60 64 68 72 76 80 84 88 92 96
Hours Post Injection
PB
UI, 5x
Phage, 41ZΔ, 3x
64LΔ
42argFΔ, 52SΔ
44GΔ
PAO1
UI
PB
PhageOnly
PAO1
41ZΔ
42argFΔ
44GΔ
52SΔ
64LΔ
3x
5x
